# Characterizing trends and associations for hepatitis C virus antibody prevalence in the Middle East and North Africa: meta-regression analyses

**DOI:** 10.1038/s41598-022-25086-5

**Published:** 2022-11-30

**Authors:** Sarwat Mahmud, Hiam Chemaitelly, Ahmed S. Alaama, Joumana G. Hermez, Laith J. Abu-Raddad

**Affiliations:** 1 Infectious Disease Epidemiology Group, Weill Cornell Medicine-, Cornell University, Qatar Foundation - Education City, P.O. Box 24144, Doha, Qatar; 2 World Health Organization Collaborating Centre for Disease Epidemiology Analytics On HIV/AIDS, Sexually Transmitted Infections, and Viral Hepatitis, Weill Cornell Medicine–Qatar, Cornell University, Qatar Foundation – Education City, Doha, Qatar; 3 Department of Communicable Diseases, HIV/Hepatitis/STIs Unit, World Health Organization Regional Office for the Eastern Mediterranean, Cairo, Egypt; 4 Department of Population Health Sciences, Weill Cornell Medicine, Cornell University, New York, NY USA; 5 Department of Public Health, College of Health Sciences, QU Health, Qatar University, Doha, Qatar

**Keywords:** Viral hepatitis, Epidemiology

## Abstract

This study characterized population-level trends and associations with hepatitis C virus (HCV) antibody (Ab) prevalence in the Middle East and North Africa (MENA). Data source was the standardized and systematically gathered MENA HCV Epidemiology Synthesis Project Database. Random-effects univariable and multivariable meta-regressions were conducted. 2,621 HCV Ab prevalence measures on 49,824,108 individuals were analyzed. In the analysis including all populations, 71% of the variation in prevalence was explained, mostly by at-risk population type. Compared to the general population, prevalence was 23-fold higher among people who inject drugs, and 14-fold higher among high-risk clinical populations. In the analysis including only the general population, 67% of the variation in prevalence was explained, mostly by country/subregion. Compared to Afghanistan, prevalence was highest in Egypt and Pakistan. Prevalence in the general population was declining at a rate of 4% per year, but outside the general population, the decline was at only 1% per year. HCV Ab prevalence in MENA is declining rapidly, but this decline is largely occurring in the general population following introduction of blood and injection safety measures. The decline in populations at higher risk of exposure is slow and below the level needed to achieve HCV elimination by 2030.

## Introduction

Viral hepatitis is the fifth leading cause of mortality in the Middle East and North Africa (MENA) region, two-thirds of which is caused by hepatitis C virus (HCV)^1,2^. MENA has the highest burden of HCV infection of all regions^1,3–5^. In 2019, an estimated 470,000 new HCV infections occurred in MENA^5^, accounting for 30% of the global number of new HCV infections^5^. Furthermore, in 2019 HCV caused 13,705 deaths due to liver cancer and 57,994 deaths due to cirrhosis and other chronic liver diseases in MENA^6^. Despite the high burden, only three MENA countries have conducted a nationally representative population-based survey to assess infection levels^7–10^. These countries include Egypt^8,10^, Pakistan^7^, and Libya^9^. HCV infection levels in the remaining countries are inadequately characterized.

With the breakthroughs in HCV treatment, and specifically, introduction of highly efficacious direct-acting antivirals (DAA)^11^, there is a historic opportunity to drastically reduce the burden of HCV infection, and even eliminate this infection as a public health concern. The World Health Organization (WHO) has set elimination of viral hepatitis as a global target by 2030^12,13^. This requires diagnosis of 90% of people living with hepatitis B and/or C and treating 80% of those eligible for treatment should be cured (hepatitis C) or virally suppressed (hepatitis B), in addition to strengthening other prevention components including hepatitis B vaccination, prevention of mother-to-child transmission of hepatitis B, injection safety and harm reduction. With this target in mind, characterizing HCV epidemiology has been rendered all the more critical to help identify carriers of this virus and treating them.

Against this background, the MENA HCV Epidemiology Synthesis Project was launched^4^, an undertaking to investigate HCV epidemiology in MENA, and to inform public health policy, programming, resource allocation, and research priorities for the region. The aim of the present study is to delineate key trends and associations in HCV epidemiology in MENA through a series of meta-regressions, using the MENA HCV Synthesis Project Database^4^ that includes approximately 2,600 systematically assembled HCV antibody (Ab) prevalence measures on 50 million individuals.

## Methods

### Data sources and database

All studies reporting HCV Ab prevalence in a MENA country were extracted from the MENA HCV Epidemiology Synthesis Project Database^4^. The database was populated through a series of systematic reviews on countries and subregions of MENA including Afghanistan^14^, Egypt^15,16^, Fertile Crescent (which includes Iraq, Jordan, Lebanon, Palestine, and Syria), Gulf (which includes Bahrain, Kuwait, Oman, Qatar, Saudi Arabia, and the United Arab Emirates)^17^, Horn of Africa (which includes Djibouti, Somalia, Sudan, and Yemen), Iran^18^, Maghreb (which includes Algeria, Libya, Mauritania, Morocco, and Tunisia)^19^, and Pakistan^20^. The database included 2,621 Ab prevalence measures on 49,824,108 tested individuals.

All reviews followed a standardized methodology^4,15–22^, informed by the Cochrane Collaboration Handbook^23^, and reported their findings using the Preferred Reporting Items for Systematic Reviews and Meta-Analysis (PRISMA)^24^. Specific methodology details, including detailed PRISMA flowcharts, can be found in each of the respective systematic reviews^4,15–22^. In brief, the data sources for these reviews included international scientific databases (PubMed and Embase), regional- and country-level databases (Iran’s Scientific Information database, Iraqi Academic Scientific Journals’ database, among others), reports and routine data from countries and international organizations, the MENA HIV/AIDs Epidemiology Synthesis Project Database^25,26^, and abstract archives of international scientific conferences.

The search criteria in each of these reviews was broad, employing a combination of index terms and free text terms for HCV and the relevant countries^4,15–22^. No language restrictions were imposed, and all records reporting HCV measures after the year it was first formally identified, 1989^27^, were included.

Informed by previous literature^28,29^, the populations tested for HCV Ab were classified into six population categories based on the level of risk of being exposed to HCV infection^4,15–22^. The categories, along with examples of these populations, can be found in Table 1. An additional category of “mixed” populations was included for studies in which populations of different levels of risk were combined and could not be separated.

**Table 1:** Population classification based on the risk of exposure to HCV infection.

Population	Definition	Examples
General population	The wider population in any country that is typically at low risk of exposure to HCV infection	Blood donors, children, pregnant women, and household-based survey participants
Populations at intermediate risk	Populations at a higher risk of exposure to HCV infection than the general population, but at a lower risk of exposure than populations at high risk	Healthcare workers, prisoners, and patients with diabetes
High-risk clinical populations	Populations at a high risk of exposure to HCV infection due to frequent exposures to blood transfusion and/or medical injections	Thalassemia, hemophilia, and hemodialysis patients
Other special clinical populations	Clinical populations in whom the risk of exposure to HCV infection is uncertain	Patients with dermatological manifestations and renal disorders
Populations with liver-related conditions	Patients with liver-related conditions that could be related to clinical disease manifestations of HCV infection	Patients with liver cirrhosis, hepatocellular carcinoma, and chronic liver disease
People who inject drugs	People who inject drugs who are at a high risk of exposure to HCV infection due to sharing of injecting equipment	-

Abbreviations: HCV, hepatitis C virus.

### Quantitative analysis

Random-effects univariable and multivariable meta-regressions were used to determine predictors and trends in HCV Ab prevalence, along with sources of between-study heterogeneity. A priori relevant variables in these analyses included population or subpopulation, country/subregion, study site, sampling methodology, sample size, year(s) of data collection, and year of publication. Variables were included in the final multivariable analysis model if they were found to be associated with HCV Ab prevalence with a p-value of ≤ 0.20 in the univariable analysis. Adjusted relative risks (ARR) were reported, quantifying the ratio of HCV Ab prevalence relative to a reference HCV Ab prevalence. An ARR with a p-value of ≤ 0.05 indicated strong evidence for an association between that variable and HCV Ab prevalence.

For studies in which the year of data collection was unavailable, this variable was imputed by subtracting the year of data collection in the rest of studies from the year of publication, and using the median of these values in imputing the year of data collection. Sensitivity analysis was performed with and without the imputed values to determine if the imputation had any impact on the results. Meta-regressions were performed on STATA version 13, using the *metan* command.

## Results

### All populations analysis

#### Main analysis

**Figure 1:**
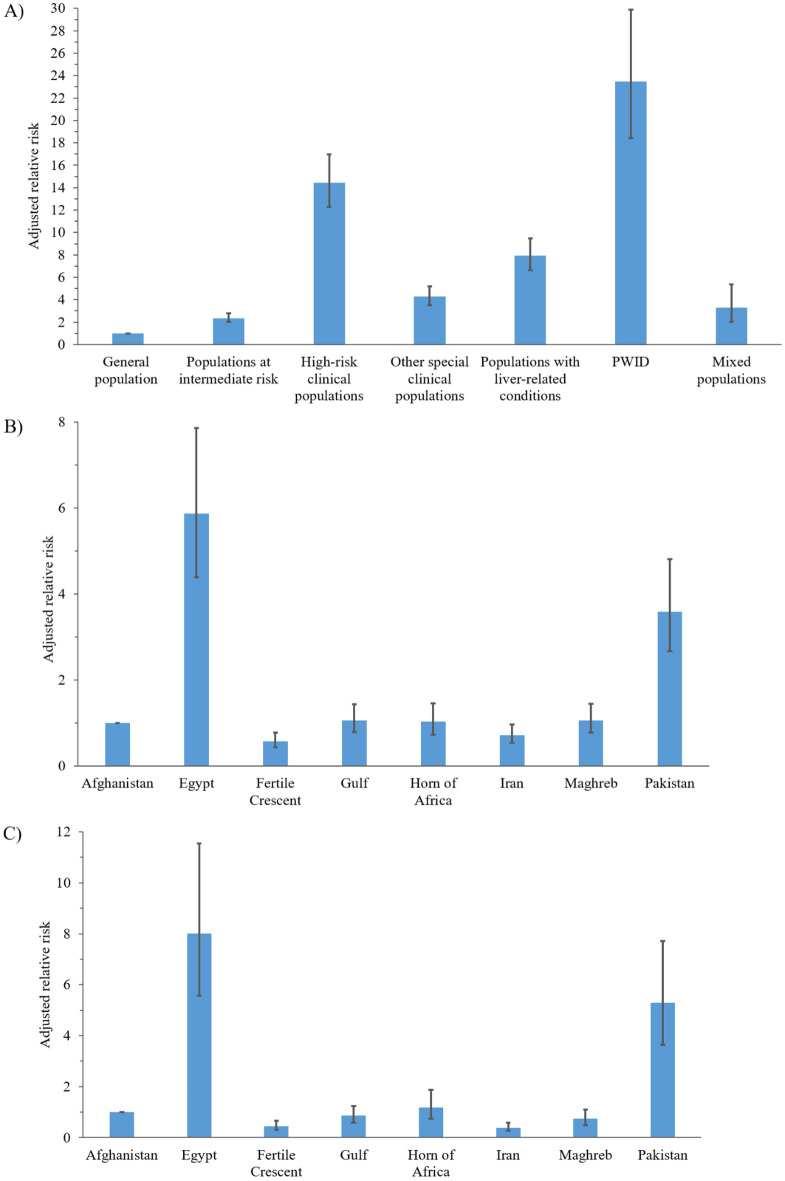
Adjusted relative risk by (**A**) population type, (**B**) country/subregion in analyses for all populations, and (**C**) country/subregion in analyses for general population.

The multivariable meta-regression analysis including all populations explained 71% of the variation in HCV Ab prevalence in MENA and showed that population type is by far the strongest predictor of prevalence (Table 2). Population type explained alone 45% of the variation in HCV Ab prevalence. There was a clear hierarchy by population type and HCV Ab prevalence was lowest in the general population (Fig. 1A). Compared to the general population, HCV Ab prevalence was highest in people who inject drugs (PWID) [ARR of 23.46, (95% confidence interval (CI):18.43–29.87, *p* < 0.001)], followed by high-risk clinical populations [ARR of 14.44, (95% CI 12.29–16.96, *p* < 0.001)] (Fig. 1A).

**Table 2:** Univariable and multivariable meta-regression analyses for HCV Ab prevalence in all populations in the Middle East and North Africa.

	Outcome measures	Sample size	Univariable analysis				Multivariable analysis^b^	
	Total N	Total n	*RR* (95% CI)	*p*-value	F *p*-value^a^	Variance explained R^2^ (%)	*ARR* (95% CI)	*p*-value
**Population characteristics**								
**Population type**								
General population	1213	48,993,158	1	–			1	–
Populations at intermediate risk	352	332,998	3.20 (2.67–3.84)	< 0.001			2.36 (2.00–2.79)	< 0.001
High-risk clinical populations	451	127,832	14.70 (12.56–17.22)	< 0.001			14.44 (12.29–16.96)	< 0.001
Other special clinical populations	215	102,148	6.52 (5.23–8.12)	< 0.001			4.27 (3.52–5.18)	< 0.001
Populations with liver-related conditions	252	132,338	15.22 (12.47–18.57)	< 0.001			7.92 (6.63–9.46)	< 0.001
PWID	118	46,602	26.28 (20.10–34.37)	< 0.001			23.46 (18.43–29.87)	< 0.001
Mixed populations	21	89,432	1.81 (0.97–3.38)	0.062	< 0.001	44.82	3.29 (2.03–5.35)	< 0.001
**Country/subregion**								
Afghanistan*	79	764,408	1	–			1	–
Egypt	474	1,727,247	8.44 (5.51–12.94)	< 0.001			5.87 (4.39–7.86)	< 0.001
Fertile Crescent^£^	445	3,785,409	0.84 (0.54–1.29)	0.416			0.58 (0.43-.78)	< 0.001
Gulf**	397	21,246,534	1.45 (0.94–2.24)	0.093			1.06 (0.79–1.43)	0.699
Horn of Africa^∞^	117	93,768	1.42 (0.85–2.40)	0.184			1.03 (0.72–1.46)	0.873
Iran	477	16,222,414	2.65 (1.72–4.07)	< 0.001			0.72 (0.54-.97)	0.029
Maghreb^¥^	218	3,614,483	1.89 (1.19–3.00)	0.007			1.06 (0.78–1.45)	0.703
Pakistan	415	2,350,245	5.59 (3.64–8.61)	< 0.001	< 0.001	17.55	3.59 (2.67–4.81)	< 0.001
**Study site**								
Blood bank	525	42,178,951	1				1	–
ANC clinic	67	68,479	3.25 (2.10–5.03)	< 0.001			2.26 (1.69–3.03)	< 0.001
Central laboratory	14	23,975	6.16 (2.50–15.18)	< 0.001			2.55 (1.39–4.67)	0.002
Clinical setting	1270	1,252,874	11.53 (9.73–13.65)	< 0.001			1.86 (1.60–2.17)	< 0.001
Community	388	4,026,291	3.89 (3.13–4.84)	< 0.001			1.75 (1.51–2.04)	< 0.001
Fertility/IVF clinic	4	2673	0.43 (0.07–2.71)	0.366			1.02 (0.28–3.72)	0.978
Military	2	182,171	0.23 (0.03–2.12)	0.195			0.37 (0.09–1.54)	0.171
Prison	72	163,893	15.54 (10.45–23.12)	< 0.001			4.79 (3.55–6.46)	< 0.001
Refugee camp	6	2,155	1.58 (0.28–8.87)	0.603			1.84 (0.55–6.19)	0.324
Rehab/drop-in-center	44	16,770	37.17 (22.71–60.85)	< 0.001			4.74 (3.28–6.84)	< 0.001
VCT	9	21,637	27.21 (9.51–77.86)	< 0.001			2.70 (1.30–5.61)	0.008
Mixed	15	3468	14.24 (6.18–32.79)	< 0.001			2.03 (1.16–3.54)	0.013
Unspecified	206	1,881,171	6.47 (4.93–8.49)	< 0.001	< 0.001	30.04	1.55 (1.27–1.90)	< 0.001
**Study methodology characteristics**								
**Sampling methodology**								
Probability-based	248	468,004	1				–	–
Nonprobability-based	2330	49,308,336	1.04 (0.80–1.34)	0.784			–	–
Unspecified	44	48,168	1.28 (0.67–2.43)	0.460	0.761	0.00	–	–
**Sample size**								
< 100	694	40,092	1				1	–
≥ 100	1928	49,784,416	0.22 (0.19–0.26)	< 0.001	< 0.001	13.80	0.71 (0.64–0.79)	< 0.001
**Temporal variation**								
Year of data collection^●^	2621	49,824,108	0.97 (0.96–0.98)	< 0.001	< 0.001	1.27	0.97 (0.96–0.98)	< 0.001
Year of publication	2621	49,824,108	0.96 (0.95–0.97)	< 0.001	< 0.001	1.78	–	–

Abbreviations: Ab, antibody; ANC, antenatal care; ARR, adjusted relative risk; CI, confidence interval; HCV, hepatitis C virus; IVF, in vitro fertilization; PWID, people who inject drugs; RR, relative risk; VCT, voluntary counseling and testing.

*Afghanistan was chosen as a reference country given the availability of considerable number of studies in the general population, low HCV prevalence in the general population, and being the first country alphabetically to qualify accordingly.

^£^Countries include Iraq, Jordan, Lebanon, Palestine, and Syria.

**Countries include Bahrain, Kuwait, Oman, Qatar, Saudi Arabia, and United Arab Emirates.

^∞^Countries include Djibouti, Somalia, Sudan, and Yemen.

^¥^Countries include Algeria, Libya, Mauritania, Morocco, and Tunisia.

^●^Due to collinearity between year of data collection and year of publication, the multivariable analysis included only one of these variables, year of data collection.

^a^Variables with a p-value ≤ 0.2 were eligible for inclusion in the multivariable analysis.

^b^The adjusted R-squared for the full model was 70.62%.

Study site was also a predictor of HCV Ab prevalence (Table 2), but largely because of the association between population type and study site. Still, after controlling for population type, testing at prisons and rehab/drop-in-centers was associated with higher HCV Ab prevalence with an ARR of 4.79 (95% CI 3.55–6.46, *p* < 0.001) and 4.74 (95% CI 3.28–6.84, *p* < 0.001), respectively, compared to testing at blood banks.

Country/subregion was a strong predictor of HCV Ab prevalence and explained alone 18% of the variation in prevalence (Table 2). HCV Ab prevalence was highest in Egypt and Pakistan (Fig. 1B). For the remaining countries/subregions, HCV Ab prevalence was much lower and the variation in prevalence across countries was small. The lowest HCV Ab prevalence levels were found in the Fertile Crescent and Iran (Fig. 1B).

The multivariable meta-regression indicated evidence that HCV Ab prevalence is declining. The rate of decline was estimated at 3% per year [ARR of 0.97, (95% CI 0.96–0.98, *p* < 0.001)].

#### Additional analysis

For a better understanding of HCV epidemiology outside the general population, the above analysis was repeated, but after excluding all measures in the general population (Table 3). The analysis confirmed similar findings for the effects of population type, study site, and country/subregion, but the estimated rate of decline in HCV Ab prevalence over the last three decades was only 1% per year [ARR of 0.99, (95% CI 0.98–1.00, *p* = 0.005)].

**Table 3:** Univariable and multivariable meta-regression analyses for HCV Ab prevalence in all populations, but excluding the general population, in the Middle East and North Africa.

	Outcome measures	Sample size	Univariable analysis				Multivariable analysis^b^	
	Total N	Total n	*RR* (95% CI)	*p*-value	F *p*-value^a^	Variance explained R^2^ (%)	*ARR* (95% CI)	*p*-value
**Population characteristics**								
**Population type**								
Populations at intermediate risk	352	332,998	1	–			1	–
High-risk clinical populations	451	127,832	4.31 (3.66–5.07)	< 0.001			5.28 (4.50–6.19)	< 0.001
Other special clinical populations	215	102,148	2.02 (1.65–2.47)	< 0.001			1.91 (1.59–2.30)	< 0.001
Populations with liver-related condition	252	132,338	4.58 (3.79–5.52)	< 0.001			3.78 (3.18–4.51)	< 0.001
PWID	118	46,602	7.52 (5.96–9.49)	< 0.001			8.59 (6.90–10.68)	< 0.001
Mixed populations	21	89,432	0.53 (0.32–0.88)	0.013	< 0.001	32.00	0.92 (0.59–1.45)	0.733
**Country/subregion**								
Afghanistan*	33	14,953	1	–			1	–
Egypt	211	69,975	3.39 (2.13–5.40)	< 0.001			4.22 (2.74–6.51)	< 0.001
Fertile Crescent^£^	224	107,001	0.84 (0.52–1.34)	0.452			0.97 (0.64–1.49)	0.901
Gulf**	156	132,326	2.01 (1.25–3.23)	0.004			2.06 (1.31–3.24)	0.002
Horn of Africa^∞^	68	16,140	0.61 (0.36–1.06)	0.080			1.13 (0.69–1.86)	0.618
Iran	354	148,935	1.39 (0.89–2.20)	0.151			1.14 (0.75–1.74)	0.533
Maghreb^¥^	127	91,233	1.37 (0.84–2.22)	0.210			1.84 (1.18–2.88)	0.007
Pakistan	236	250,787	2.26 (1.42–3.58)	< 0.001	< 0.001	12.94	3.29 (2.15–5.04)	< 0.001
**Study site**								
Blood bank	17	47,443	1				1	–
Clinical setting	1021	475,579	3.96 (1.75–8.98)	< 0.001			1.71 (1.08–2.71)	0.022
Community	102	25,267	2.35 (1.00–5.56)	0.051			1.53 (0.93–2.54)	0.097
Prison	72	103,102	4.00 (1.68–9.52)	0.002			3.96 (2.36–6.64)	< 0.001
Rehab/drop-in-center	43	16,736	8.65 (3.52–21.25)	< 0.001			3.53 (2.05–6.09)	< 0.001
VCT	8	1,762	8.31 (2.48–27.83)	< 0.001			2.82 (1.20–6.65)	0.018
Mixed	10	1,033	3.88 (1.22–12.38)	0.022			1.37 (0.65–2.90)	0.408
Unspecified	136	160,428	3.23 (1.39–7.54)	0.007	< 0.001	2.95	1.67 (1.01–2.74)	0.044
**Study methodology characteristics**								
**Sampling methodology**								
Probability-based	66	21,367	1				1	–
Nonprobability-based	1316	797,620	1.45 (1.03–2.03)	0.034			1.05 (0.81–1.36)	0.703
Unspecified	27	12,363	1.50 (0.82–2.75)	0.186	0.102	0.28	1.30 (0.85–2.00)	0.231
**Sample size**								
< 100	555	32,034	1				1	–
≥ 100	854	799,316	0.60 (0.52–0.69)	< 0.001	< 0.001	4.92	0.77 (0.69–0.86)	< 0.001
**Temporal variation**								
Year of data collection^●^	1408	831,350	0.97 (0.96–0.98)	< 0.001	< 0.001	2.34	0.99 (0.98–1.00)	0.005
Year of publication	1408	831,350	0.97 (0.96–0.99)	< 0.001	< 0.001	2.07	–	–

Abbreviations: Ab, antibody; ARR, adjusted relative risk; CI, confidence interval; HCV, hepatitis C virus; PWID, people who inject drugs; RR, relative risk; VCT, voluntary counseling and testing.

*Afghanistan was chosen as a reference country given the availability of considerable number of studies in the general population, low HCV prevalence in the general population, and being the first country alphabetically to qualify accordingly.

^£^Countries include Iraq, Jordan, Lebanon, Palestine, and Syria.

**Countries include Bahrain, Kuwait, Oman, Qatar, Saudi Arabia, and United Arab Emirates.

^∞^Countries include Djibouti, Somalia, Sudan, and Yemen.

^¥^Countries include Algeria, Libya, Mauritania, Morocco, and Tunisia.

^●^Due to collinearity between year of data collection and year of publication, the multivariable analysis included only one of these variables, year of data collection.

^a^Variables with a p-value ≤ 0.2 were eligible for inclusion in the multivariable analysis.

^b^The adjusted R-squared for the full model was 51.54%.

### General population analysis

#### Main analysis

The analysis including only the general population explained 67% of the variation in HCV Ab prevalence in MENA and showed that country/subregion is by far the strongest predictor of prevalence (Table 4). Country/subregion explained alone 58% of the variation in HCV Ab prevalence. Compared to Afghanistan as a reference country, HCV Ab prevalence in the general population was highest in Egypt [ARR of 8.02, (95% CI 5.57–11.55, *p* < 0.001)] and Pakistan [ARR of 5.30, (95% CI 3.64–7.72, *p* < 0.001)]. For the remaining countries/subregions, HCV Ab prevalence was much lower and the variation in prevalence across countries was small. The lowest HCV Ab prevalence levels were found in the Fertile Crescent and Iran (Fig. 1C).

**Table 4:** Univariable and multivariable meta-regression analyses for HCV Ab prevalence in the general population in the Middle East and North Africa.

	Outcome measures	Sample size	Univariable analysis				Multivariable analysis^b^	
	Total N	Total n	*RR* (95% CI)	*p*-value	F *p*-value^a^	Variance explained R^2^ (%)	*ARR* (95% CI)	*p*-value
**Population characteristics**								
**Subpopulation type**								
Blood donors	686	46,646,973	1				1	–
Community members	300	1,750,188	3.77 (3.01–4.73)	< 0.001			2.00 (1.55–2.59)	< 0.001
Children	45	20,279	1.80 (1.02–3.19)	0.042			0.76 (0.50–1.15)	0.191
Refugees/asylum seekers	8	3279	2.20 (0.56–8.62)	0.255			3.01 (0.70–12.91)	0.138
Pregnant women	71	70,577	2.92 (1.91–4.45)	< 0.001			0.72 (0.17–3.05)	0.655
Military/army recruits	18	259,742	1.71 (0.80–3.65)	0.163			0.88 (0.52–1.50)	0.640
Outpatient attendees	22	42,801	6.25 (3.08–12.68)	< 0.001			2.90 (1.79–4.70)	< 0.001
Pre-employment/martial screening	39	131,608	1.05 (0.62–1.80)	0.852			1.21 (0.75–1.95)	0.427
Couples seeking fertility treatment	6	3,597	0.43 (0.09–2.05)	0.290			1.17 (0.16–8.90)	0.876
College students	11	19,075	0.52 (0.18–1.51)	0.227			0.70 (0.33–1.48)	0.349
Other general populations	7	45,039	4.66 (1.38–15.74)	0.013	< 0.001	13.54	2.00 (0.89–4.52)	0.095
**Country/subregion**								
Afghanistan*	46	749,455	1	–			1	–
Egypt	263	1,677,272	14.89 (10.18–21.78)	< 0.001			8.02 (5.57–11.55)	< 0.001
Fertile Crescent^£^	221	3,678,408	0.52 (0.35–0.77)	< 0.001			0.45 (0.31–0.65)	< 0.001
Gulf**	241	21,114,208	1.24 (0.84–1.82)	0.280			0.85 (0.59–1.23)	0.398
Horn of Africa^∞^	49	77,628	2.05 (1.25–3.37)	0.005			1.18 (0.74–1.88)	0.490
Iran	123	16,073,479	0.50 (0.33–0.77)	< 0.001			0.39 (0.26–0.58)	< 0.001
Maghreb^¥^	91	3,523,250	1.02 (0.66–1.56)	0.936			0.74 (0.49–1.10)	0.139
Pakistan	179	2,099,458	6.96 (4.71–10.29)	< 0.001	< 0.001	58.39	5.30 (3.64–7.72)	< 0.001
**Study site**								
Blood bank	515	42,193,774	1	–			1	–
ANC clinic	67	68,479	3.38 (2.17–5.26)	< 0.001			2.48 (0.57–10.84)	0.227
Central laboratory	14	23,975	6.41 (2.57–15.99)	< 0.001			1.50 (0.71–3.18)	0.285
Clinical setting	243	775,854	2.38 (1.82–3.11)	< 0.001			1.16 (0.93–1.43)	0.182
Community	286	4,001,024	3.06 (2.40–3.90)	< 0.001			0.79 (0.60–1.04)	0.095
Fertility/IVF clinic	4	2,673	0.44 (0.07–2.87)	0.391			1.56 (0.14–17.01)	0.717
Military	2	182,171	0.24 (0.03–2.30)	0.216			0.41 (0.09–1.80)	0.235
Refugee camp	6	2,155	1.60 (0.28–9.11)	0.597			0.49 (0.08–3.21)	0.459
VCT	1	19,875	3.21 (0.14–76.54)	0.47			4.97 (0.70–35.22)	0.108
Mixed	5	2,435	10.95 (2.57–46.57)	< 0.001			1.88 (0.74–4.77)	0.185
Unspecified	70	1,720,743	1.64 (1.07–2.53)	0.024	< 0.001	10.02	1.25 (0.93–1.69)	0.142
**Study methodology characteristics**								
**Sampling methodology**								
Probability-based	182	446,637	1				1	–
Nonprobability-based	1014	48,510,716	0.34 (0.26–0.45)	< 0.001			0.65 (0.54–0.78)	< 0.001
Unspecified	17	35,805	0.22 (0.08–0.59)	0.003	< 0.001	5.21	0.93 (0.49–1.78)	0.825
**Sample size**								
< 100	139	8,058	1				1	–
≥ 100	1074	48,985,100	0.21 (0.15–0.29)	< 0.001	< 0.001	8.31	0.60 (0.47–0.77)	< 0.001
**Temporal variation**								
Year of data collection^●^	1213	48,993,158	0.95 (0.93–0.96)	< 0.001	< 0.001	4.35	0.96 (0.95–0.97)	< 0.001
Year of publication	1213	48,993,158	0.93 (0.92–0.95)	< 0.001	< 0.001	6.92	–	–

Abbreviations: Ab, antibody; ANC, antenatal care; ARR, adjusted relative risk; CI, confidence interval; HCV, hepatitis C virus; RR, relative risk; VCT, voluntary counseling and testing.

*Afghanistan was chosen as a reference country given the availability of considerable number of studies in the general population, low HCV prevalence in the general population, and being the first country alphabetically to qualify accordingly.

^£^Countries include Iraq, Jordan, Lebanon, Palestine, and Syria.

**Countries include Bahrain, Kuwait, Oman, Qatar, Saudi Arabia, and United Arab Emirates.

^∞^Countries include Djibouti, Somalia, Sudan, and Yemen.

^¥^Countries include Algeria, Libya, Mauritania, Morocco, and Tunisia.

^●^Due to collinearity between year of data collection and year of publication, the multivariable analysis included only one of these variables, year of data collection.

^a^Variables with a p-value ≤ 0.2 were eligible for inclusion in the multivariable analysis.

^b^The adjusted R-squared for the full model was 66.62%.

There were only small differences in HCV Ab prevalence by subpopulation of the general population and by study site (Table 4). Most of the differences did not reach statistical significance. There was evidence that HCV Ab prevalence in the general population is declining. The rate of decline was estimated at 4% per year [ARR of 0.96, (95% CI 0.95–0.97), *p* < 0.001)].

#### Additional analysis

To confirm the findings of the above analysis, the analysis was repeated excluding all measures in blood donors (Table 5). This was conducted as blood donor data may lead to underestimation of HCV prevalence in the general population at large^30,31^—blood donors tend to be selectively a healthy and low risk population that is less likely to be HCV infected^30^. The analysis confirmed similar findings for the effects of country/subregion, subpopulation type, and study site, but the estimated rate of decline in HCV Ab prevalence was slightly lower—it was estimated at 3% per year [ARR of 0.97, (95% CI 0.96–0.98), *p* < 0.001)].

**Table 5:** Univariable and multivariable meta-regression analyses for HCV Ab prevalence in the general population, but excluding blood donors, in the Middle East and North Africa.

	Outcome measures	Sample size	Univariable analysis				Multivariable analysis^b^	
	Total N	Total n	*RR* (95% CI)	*p*-value	F *p*-value^a^	Variance explained R^yyyyy^ (%)	*ARR* (95% CI)	*p*-value
**Population characteristics**								
**Subpopulation type**								
Community members	300	1,750,188	1	–			1	–
Children	45	20,279	0.48 (0.28–0.85)	0.011			0.38 (0.26–0.56)	< 0.001
Refugees/asylum seekers	8	3,279	0.61 (0.17–2.23)	0.453			1.18 (0.29–4.81)	0.814
Pregnant women	71	70,577	0.78 (0.51–1.18)	0.239			0.29 (0.07–1.14)	0.076
Military/army recruits	18	259,742	0.45 (0.22–0.93)	0.030			0.38 (0.23–0.65)	< 0.001
Outpatient attendees	22	42,801	1.66 (0.84–3.27)	0.145			1.33 (0.83–2.14)	0.230
Pre-employment/martial screening	39	131,608	0.28 (0.16–0.47)	< 0.001			0.49 (0.32–0.77)	0.002
Couples seeking fertility treatment	6	3,597	0.12 (0.03–0.51)	0.005			0.48 (0.07–3.22)	0.447
College students	11	19,075	0.14 (0.05–0.38)	< 0.001			0.30 (0.15–0.59)	< 0.001
Other general populations	7	45,039	1.23 (0.39–3.90)	0.726	< 0.001	8.61	0.76 (0.36–1.61)	0.474
**Country/subregion**								
Afghanistan*	6	12,048	1	–			1	
Egypt	147	110,603	12.59 (4.76–33.27)	< 0.001			5.87 (2.16–16.00)	< 0.001
Fertile Crescent^£^	64	189,456	0.70 (0.26–1.93)	0.492			0.42 (0.15–1.18)	0.100
Gulf**	85	222,829	1.18 (0.44–3.17)	0.740			0.84 (0.30–2.31)	0.730
Horn of Africa^∞^	27	29,552	2.22 (0.78–6.37)	0.136			1.05 (0.36–3.05)	0.928
Iran	50	101,677	0.67 (0.24–1.88)	0.452			0.38 (0.13–1.10)	0.074
Maghreb^¥^	42	1,378,206	0.91 (0.33–2.52)	0.855			0.53 (0.19–1.47)	0.223
Pakistan	106	301,814	7.77 (2.93–20.63)	< 0.001	< 0.001	57.46	5.17 (1.92–13.97)	< 0.001
**Study site**								
Community	265	1,868,697	1	–			1	–
ANC clinic	67	68,479	1.10 (0.71–1.72)	0.664			3.59 (0.90–14.37)	0.071
Central laboratory	14	23,975	2.09 (0.86–5.06)	0.102			2.55 (1.30–4.99)	0.006
Clinical setting	138	164,592	1.08 (0.76–1.54)	0.676			1.49 (1.15–1.93)	0.003
Fertility/IVF clinic	4	2,673	0.15 (0.02–0.88)	0.036			1.97 (0.20–19.20)	0.559
Military	2	175,322	0.08 (0.01–0.67)	0.020			0.56 (0.13–2.40)	0.435
Refugee camp	6	2,155	0.55 (0.10–2.94)	0.482			0.67 (0.11–3.96)	0.657
Mixed	4	2,335	4.94 (1.08–22.64)	0.040			1.94 (0.77–4.86)	0.157
Unspecified	72	31,108	1.71 (0.87–3.37)	0.117	0.011	2.72	2.32 (1.44–3.72)	< 0.001
**Study methodology characteristics**								
**Sampling methodology**								
Probability-based	127	369,759	1				1	–
Nonprobability-based	388	1,971,157	0.80 (0.57–1.11)	0.176			0.74 (0.59–0.92)	0.007
Unspecified	12	5,269	0.28 (0.09–0.89)	0.032	0.065	0.20	0.89 (0.41–1.93)	0.760
**Sample size**								
< 100	94	5298	1				1	–
≥ 100	433	2,340,887	0.35 (0.23–0.53)	< 0.001	< 0.001	7.78	0.66 (0.49–0.88)	0.006
**Temporal variation**								
Year of data collection^●^	527	2,346,185	0.96 (0.94–0.98)	< 0.001	< 0.001	3.38	0.97 (0.96–0.98)	< 0.001
Year of publication	527	2,346,185	0.95 (0.93–0.97)	< 0.001	< 0.001	4.97	–	–

Abbreviations: Ab, antibody; ANC, antenatal care; ARR, adjusted relative risk; CI, confidence interval; HCV, hepatitis C virus; IVF, in vitro fertilization; RR, relative risk.

*Afghanistan was chosen as a reference country given the availability of considerable number of studies in the general population, low HCV prevalence in the general population, and being the first country alphabetically to qualify accordingly.

^£^Countries include Iraq, Jordan, Lebanon, Palestine, and Syria.

**Countries include Bahrain, Kuwait, Oman, Qatar, Saudi Arabia, and United Arab Emirates.

^∞^Countries include Djibouti, Somalia, Sudan, and Yemen.

^¥^Countries include Algeria, Libya, Mauritania, Morocco, and Tunisia.

^●^Due to collinearity between year of data collection and year of publication, the multivariable analysis included only one of these variables, year of data collection.

^a^Variables with a p-value ≤ 0.2 were eligible for inclusion in the multivariable analysis.

^b^The adjusted R-squared for the full model was 66.39%.

### HCV Ab prevalence and study methods

There was evidence for a strong small-study effect in all analyses. HCV Ab prevalence was lower in studies with a sample size ≥ 100 participants. In the analysis including all populations (Table 2), studies with a sample size ≥ 100 participants reported 29% lower prevalence than studies with a sample size < 100 [ARR of 0.71, (95% CI 0.64–0.79), *p* < 0.001], indicating a small-study effect. In the analysis for the general population (Table 4), studies with a sample size ≥ 100 participants reported 40% lower prevalence than studies with a sample size < 100 [ARR of 0.60, (95% CI 0.47–0.77), *p* < 0.001], indicating also a small-study effect.

There was evidence that the sampling methodology also had an effect on reported HCV Ab prevalence, but only in the general population (Table 4). Studies in the general population that did not use probability-based sampling reported 35% lower prevalence than studies that used probability-based sampling [ARR of 0.65, (95% CI 0.54–0.78), *p* < 0.001]. No evidence was seen for this effect in the analysis for all populations other than the general population (Table 3).

A sensitivity analysis was conducted to examine whether the imputation calculation for the year of data collection could have affected the study findings. The analysis confirmed the same findings for both, the analysis for all populations (Supplementary Table S1) and the analysis for the general population (Supplementary Table S2). An additional sensitivity analysis was conducted excluding studies with a sample size of < 100 participants, to examine the impact of the small-study effect that was observed in the previous analyses. The analysis confirmed the same findings as those of the main analysis (Supplementary Table S3).

## Discussion

HCV Ab prevalence is declining rapidly in the population of MENA at a rate of 3–4% per year (Tables 2 and 4). This decline may be explained by the broad improvements in blood supply screening, injection safety, and infection control that have been rolled out over the last three decades following the discovery of this infection^32^. The observed decline supports the effectiveness of these interventions and demonstrates the importance of their scale-up in all countries. Demography may also contribute to explaining the decline. The decline may reflect a cohort effect, in a context of rapidly growing population size over the last three decades^33,34^. Exposure to HCV infection may have been higher in earlier decades^34^, but most of the living population of MENA was born after the year 1990.

However, this decline largely reflects the decline in HCV transmission in the general population, where the rate of decline was steepest (Tables 4 and 5). The decline in populations at higher risk of exposure was modest at only 1% per year (Table 3). This suggests that HCV transmission is increasingly becoming concentrated in higher risk populations with limited transmission in the general population, at least in most countries. This is a consequence of the specific interventions that have been effectively implemented in MENA, including injection and blood safety^32^, that reduced transmission in the general population, but less so in other populations, including PWID and high-risk clinical populations. This highlights the importance of expanding harm reduction interventions among PWID^35,36^ and in prisons^37^, and the need for further improvements in infection control at healthcare facilities^38^.

The findings indicate reasons for optimism, but also reasons for concern. The rapidly declining HCV Ab prevalence is consistent with progress towards the WHO elimination target by 2030. However, the findings also highlight that this progress is likely to stall, as the improvements are happening in the general population and not in the populations at higher risk, where most infection incidence will be occurring over the coming years. Without major improvements in tackling HCV incidence among PWID and high-risk clinical populations, such as through different strategies for screening and treatment^39–41^, it is unlikely that the region will achieve the WHO elimination target by 2030. Moreover, improvements may materialize in some countries of the region, such as in Egypt, which has had a successful national program for screening and treatment^42^, but less so in other countries such as in Pakistan where incidence continues at considerable levels, including in the general population^43–46^.

The findings demonstrate a clear epidemiological pattern of a hierarchy in HCV Ab prevalence by population type. The likelihood of being infected varied immensely across populations. Compared to the general population, the risk of being infected was 23-fold higher for PWID and 14-fold higher for high-risk clinical populations (Table 2). HCV Ab prevalence varied also substantially across countries/subregions within MENA, highlighting how the epidemiology of this infection can vary from one country to another, even within the same region, reflecting historical factors, particularly relating to expansion of healthcare during the twentieth century^43,44,47,48^.

The analyses identified a strong small-study effect^49^ in reporting HCV Ab prevalence (Tables 2–5). For example, in studies among the general population, studies with a sample size ≥ 100 participants reported 40% lower prevalence than studies with a sample size < 100, highlighting how studies employing small samples are likely to have been in select populations that were not in truth representative of the wider general population.

The analyses identified also a strong effect for the sampling methodology on reported HCV Ab prevalence, but only in the general population (Tables 4 and 5). Studies in the general population that did not use probability-based sampling reported 35% lower prevalence than studies that used probability-based sampling. This outcome may reflect that studies using convenience samples drawn from the general population are likely to underestimate HCV Ab prevalence in the total population, perhaps because of under-sampling of PWID and high-risk clinical populations who are harder to reach and may not participate in general population surveys^50^.

This study aimed to characterize trends and associations with HCV Ab prevalence, however, not all those who are HCV Ab positive are chronically infected by HCV. Our earlier review and meta-analysis for HCV viremic rate (proportion of chronically infected individuals out of HCV Ab positive individuals) found that the overall pooled mean viremic rate in MENA is 67.6% (95% CI 64.9–70.3%)^51^. Across risk populations, the pooled mean rate ranges between 57.4% (95% CI 49.4–65.2%) in people who inject drugs, and 75.5% (95% CI 61.0–87.6%) in populations with liver-related conditions^51^. Across countries/subregions of MENA, the pooled mean rate ranges between 62.1% (95% CI 50.0–72.7%) and 70.4% (95% CI 65.5–75.1%)^51^.

Recent scale-up of HCV treatment, such as in Egypt^42^, should also have reduced the viremic rate in at least some countries. These reductions in viremic rate, that are coincident with HCV Ab prevalence declines, should amplify the reductions in HCV incidence in MENA. This outcome highlights the need to have current estimates for HCV incidence in the different countries of the region, as well as estimates of those chronically infected and in need of treatment, to inform policy and programming efforts. Such estimates can be available through mathematical modeling, as was done for Egypt^52^ and Pakistan^41^, but such studies need to be extended also to the rest of the MENA countries.

This study has limitations. With the scale-up of HCV treatment, HCV Ab prevalence will increasingly become less associated with chronic-infection prevalence^51,53^. However, the impact of this on presented analyses is probably minimal given that treatment scale-up is recent in MENA and treatment coverage remains low in nearly all countries in the region^54^. There was variability in the availability of data across MENA countries. Despite this, a key strength of this study is the large analyzed database, assembled through a standardized protocol, which to our knowledge, is the largest and most comprehensive for HCV infection in MENA. The use of this database allowed for extensive analyses in the present study that should provide in the future a framework and quantitative adjustment factors for a representative mathematical modeling estimation of HCV infection levels and numbers of persons affected by this infection in each of MENA countries. The study provides a methodological component in the estimation processes of HCV infection and disease burden at a time when these estimates are critically needed to track and validate progress towards HCV elimination by 2030.

In conclusion, HCV Ab prevalence is declining in MENA, however, this decline is mainly occurring in the general population, due to interventions such as injection and blood safety. Despite this decline, HCV incidence remains higher in MENA relative to other WHO regions, with an estimated 470,000 new HCV infections in 2019^5^. HCV infection appears to be increasingly concentrated in higher risk populations. Achieving elimination of HCV infection by 2030 hinges on expansion of screening and treatment programs, harm reduction services for PWID in settings such as prisons and rehab/drop-in-centers, and enforcement of stringent infection control and sanitary healthcare practices in clinical settings.

## Supplementary Information

Supplementary Information.

## Data Availability

The data analyzed during this study were previously published in a series of systematic reviewes^14–22^.
