# Supplementary material for: Characterizing trends and associations for hepatitis C virus antibody prevalence in the Middle East and North Africa: meta-regression analyses
Source: Sci Rep. 2022 Nov 30;12:20637. doi: 10.1038/s41598-022-25086-5 (PMC9712517; doi:10.1038/s41598-022-25086-5)
Supplement: Supplementary file 1 — Supplementary Information. [file 41598_2022_25086_MOESM1_ESM.docx]

**SUPPLEMENTARY MATERIAL**

**Table S1.** Univariable and multivariable meta-regression analyses for HCV Ab prevalence in all populations, but excluding studies with missing date of data collection, in the Middle East and North Africa.

|  |  | **Outcome measures** | **Sample size** | **Univariable analysis** | | | | **Multivariable analysis^b^** | |
| --- | --- | --- | --- | --- | --- | --- | --- | --- | --- |
|  |  | **Total N** | **Total n** | ***RR***  **(95% CI)** | **p-value** | **F p-value**^a^ | **Variance explained R^2^ (%)** | ***ARR***  **(95% CI)** | **p-value** |
| **Population characteristics** | **Population type** |  |  |  |  |  |  |  |  |
|  | General population | 1,005 | 48,346,130 | 1 | - |  |  | 1 | - |
|  | Populations at intermediate risk | 276 | 313,510 | 3.33 (2.71-4.09) | <0.001 |  |  | 2.62 (2.17-3.16) | <0.001 |
|  | High-risk clinical populations | 341 | 113,736 | 15.31 (12.78-18.35) | <0.001 |  |  | 17.15 (14.20-20.72) | <0.001 |
|  | Other special clinical populations | 160 | 94,130 | 7.55 (5.88-9.71) | <0.001 |  |  | 5.05 (4.05-6.29) | <0.001 |
|  | Populations with liver-related conditions | 181 | 119,784 | 16.54 (13.11-20.88) | <0.001 |  |  | 9.00 (7.31-11.09) | <0.001 |
|  | PWID | 98 | 41,461 | 31.41 (23.35-42.24) | <0.001 |  |  | 28.72 (22.01-37.48) | <0.001 |
|  | Mixed populations | 16 | 63,020 | 1.66 (0.81-3.41) | 0.170 | <0.001 | 45.77 | 3.37 (1.92-5.93) | <0.001 |
|  | **Country/subregion** |  |  |  |  |  |  |  |  |
|  | Afghanistan^*^ | 78 | 764,329 | 1 | - |  |  | 1 | - |
|  | Egypt | 309 | 1,709,754 | 7.03 (4.47-11.06) | <0.001 |  |  | 6.63 (4.89-8.99) | <0.001 |
|  | Fertile Crescent^£^ | 385 | 3,748,376 | 0.76 (0.49-1.19) | 0.234 |  |  | 0.56 (0.41-0.75) | <0.001 |
|  | Gulf^**^ | 301 | 21,184,281 | 1.18 (0.75-1.86) | 0.481 |  |  | 1.03 (0.76-1.40) | 0.847 |
|  | Horn of Africa^∞^ | 100 | 75,733 | 1.42 (0.82-2.47) | 0.212 |  |  | 1.06 (0.73-1.53) | 0.761 |
|  | Iran | 390 | 16,147,645 | 2.32 (1.49-3.63) | <0.001 |  |  | 0.67 (0.50-0.91) | 0.010 |
|  | Maghreb^¥^ | 170 | 20,154 | 1.72 (1.05-2.80) | 0.031 |  |  | 1.08 (0.78-1.49) | 0.636 |
|  | Pakistan | 344 | 2,035,430 | 5.48 (3.50-8.58) | <0.001 | <0.001 | 16.42 | 3.64 (2.69-4.93) | <0.001 |
|  | **Study site** |  |  |  |  |  |  |  |  |
|  | Blood bank | 485 | 41,865,252 | 1 |  |  |  | 1 | - |
|  | ANC clinic | 49 | 55,344 | 3.24 (1.94-5.39) | <0.001 |  |  | 2.26 (1.60-3.18) | <0.001 |
|  | Central laboratory | 6 | 18,993 | 1.31 (0.30-5.79) | 0.722 |  |  | 2.76 (0.97-7.84) | 0.056 |
|  | Clinical setting | 973 | 1,173,727 | 11.85 (9.88-14.21) | <0.001 |  |  | 1.72 (1.45-2.05) | <0.001 |
|  | Community | 312 | 3,912,108 | 3.78 (2.98-4.78) | <0.001 |  |  | 1.70 (1.44-2.00) | <0.001 |
|  | Fertility/IVF clinic | 4 | 2,673 | 0.47 (0.07-2.96) | 0.418 |  |  | 1.15 (0.31-4.22) | 0.831 |
|  | Military | 2 | 182,171 | 0.25 (0.03-2.32) | 0.224 |  |  | 0.39 (0.09-1.63) | 0.196 |
|  | Prison | 60 | 159,283 | 17.36 (11.27-26.74) | <0.001 |  |  | 4.44 (3.20-6.16) | <0.001 |
|  | Refugee camp | 3 | 638 | 6.73 (0.81-56.25) | 0.078 |  |  | 3.73 (0.91-15.36) | 0.068 |
|  | Rehab/drop-in-center | 35 | 14,553 | 40.17 (23.19-69.60) | <0.001 |  |  | 4.45 (2.97-6.69) | <0.001 |
|  | VCT | 9 | 21,637 | 29.78 (10.39-85.34) | <0.001 |  |  | 2.40 (1.15-5.02) | 0.020 |
|  | Mixed | 6 | 2,628 | 18.77 (5.18-68.01) | <0.001 |  |  | 4.25 (1.84-9.81) | <0.001 |
|  | Unspecified | 133 | 1,682,764 | 4.73 (3.43-6.52) | <0.001 | <0.001 | 32.07 | 1.61 (1.26-2.05) | <0.001 |
| **Study methodology characteristics** | **Sampling methodology** |  |  |  |  |  |  |  |  |
|  | Probability-based | 204 | 373,945 | 1 |  |  |  | - | - |
|  | Nonprobability-based | 1,852 | 48,682,492 | 0.79 (0.60-1.06) | 0.113 |  |  | - | - |
|  | Unspecified | 21 | 35,334 | 0.98 (0.39-2.48) | 0.971 | 0.261 | 0.03 | - | - |
|  | **Sample size** |  |  |  |  |  |  |  |  |
|  | <100 | 461 | 27,082 | 1 |  |  |  | 1 | - |
|  | ≥100 | 1,616 | 49,064,689 | 0.22 (0.18-0.27) | <0.001 | <0.001 | 11.51 | 0.69 (0.60-0.78) | <0.001 |
| **Temporal variation** | **Year of data collection^●^** | 2,077 | 49,091,771 | 0.97 (0.96-0.99) | <0.001 | <0.001 | 0.75 | 0.97 (0.96-0.98) | <0.001 |
|  | **Year of publication** | 2,077 | 49,091,771 | 0.97 (0.95-0.98) | <0.001 | <0.001 | 1.14 | - | - |

Abbreviations: Ab, antibody; ANC, antenatal care; ARR, adjusted relative risk; CI, confidence interval; HCV, hepatitis C virus; IVF, in vitro fertilization; PWID, people who inject drugs; RR, relative risk; VCT, voluntary counselling and testing.

*Afghanistan was chosen as a reference country given the availability of studies in the general population, low prevalence, and being the first country alphabetically to qualify accordingly.

^£^Countries include Iraq, Jordan, Lebanon, Palestine, and Syria.

^**^Countries include Bahrain, Kuwait, Oman, Qatar, Saudi Arabia, and United Arab Emirates.

^∞^Countries include Djibouti, Somalia, Sudan, and Yemen.

^¥^Countries include Algeria, Libya, Mauritania, Morocco, and Tunisia.

**^●^**Due to collinearity between year of data collection and year of publication, the multivariable analysis included only one of these variables, year of data collection.

^a^Variables with a p-value ≤ 0.2 were eligible for inclusion in the multivariable analysis.

^b^The adjusted R-squared for the full model was 71.58%.

**Table S2.** Univariable and multivariable meta-regression analyses for HCV Ab prevalence in the general population, but excluding studies with missing date of data collection, in the Middle East and North Africa.

|  |  | **Outcome measures** | **Sample size** | **Univariable analysis** | | | | **Multivariable analysis^b^** | |
| --- | --- | --- | --- | --- | --- | --- | --- | --- | --- |
|  |  | **Total N** | **Total n** | ***RR***  **(95% CI)** | **p-value** | **F p-value**^a^ | **Variance explained R^2^ (%)** | ***ARR***  **(95% CI)** | **p-value** |
| **Population characteristics** | **Subpopulation type** |  |  |  |  |  |  |  |  |
|  | Blood donors | 607 | 46,136,607 | 1 |  |  |  | 1 | - |
|  | Community members | 228 | 1,650,626 | 3.78 (2.95-4.86) | <0.001 |  |  | 2.23 (1.64-3.02) | <0.001 |
|  | Children | 28 | 10,838 | 2.42 (1.20-4.91) | 0.014 |  |  | 0.92 (0.54-1.56) | 0.758 |
|  | Refugees/asylum seekers | 5 | 1,762 | 5.68 (1.25-25.87) | 0.025 |  |  | 3.45 (0.79-15.09) | 0.100 |
|  | Pregnant women | 52 | 55,906 | 2.89 (1.77-4.72) | <0.001 |  |  | 2.02 (0.24-17.16) | 0.520 |
|  | Military/army recruits | 14 | 258,722 | 1.02 (0.44-2.37) | 0.959 |  |  | 0.66 (0.35-1.22) | 0.184 |
|  | Outpatient attendees | 18 | 38,507 | 8.39 (4.00-17.61) | <0.001 |  |  | 3.17 (1.89-5.34) | <0.001 |
|  | Pre-employment/martial screening | 30 | 125,751 | 0.55 (0.30-1.02) | 0.057 |  |  | 1.50 (0.90-2.51) | 0.118 |
|  | Couples seeking fertility treatment | 6 | 3,597 | 0.48 (0.10-2.24) | 0.351 |  |  | 1.31 (0.17-10.13) | 0.796 |
|  | College students | 10 | 18,775 | 0.60 (0.20-1.80) | 0.359 |  |  | 0.86 (0.39-1.92) | 0.717 |
|  | Other general populations | 7 | 45,039 | 5.17 (1.56-17.16) | 0.007 | <0.001 | 15.05 | 2.11 (0.91-4.91) | 0.084 |
|  | **Country/subregion** |  |  |  |  |  |  |  |  |
|  | Afghanistan^*^ | 46 | 749,455 | 1 | - |  |  | 1 | - |
|  | Egypt | 199 | 1,651,555 | 13.24 (8.97-19.56) | <0.001 |  |  | 8.21 (5.64-11.94) | <0.001 |
|  | Fertile Crescent^£^ | 194 | 3,645,078 | 0.48 (0.32-0.72) | <0.001 |  |  | 0.45 (0.31-0.65) | <0.001 |
|  | Gulf^**^ | 197 | 21,058,714 | 1.10 (0.74-1.62) | 0.650 |  |  | 0.79 (0.55-1.16) | 0.228 |
|  | Horn of Africa^∞^ | 40 | 61,428 | 2.18 (1.29-3.69) | 0.004 |  |  | 1.28 (0.78-2.10) | 0.336 |
|  | Iran | 108 | 16,011,868 | 0.48 (0.31-0.74) | <0.001 |  |  | 0.38 (0.25-0.57) | <0.001 |
|  | Maghreb^¥^ | 72 | 3,366,696 | 0.99 (0.64-1.55) | 0.978 |  |  | 0.76 (0.50-1.17) | 0.212 |
|  | Pakistan | 149 | 1,801,336 | 6.50 (4.36-9.69) | <0.001 | <0.001 | 57.34 | 5.12 (3.47-7.57) | <0.001 |
|  | **Study site** |  |  |  |  |  |  |  |  |
|  | Blood bank | 478 | 41,880,227 | 1 | - |  |  | 1 | - |
|  | ANC clinic | 49 | 55,344 | 3.31 (1.98-5.54) | <0.001 |  |  | 0.94 (0.11-8.19) | 0.952 |
|  | Central laboratory | 6 | 18,993 | 1.34 (0.30-5.98) | 0.698 |  |  | 1.97 (0.64-6.05) | 0.236 |
|  | Clinical setting | 177 | 741,220 | 2.23 (1.65-2.99) | <0.001 |  |  | 1.12 (0.88-1.42) | 0.353 |
|  | Community | 237 | 3,889,904 | 3.11 (2.40-4.03) | <0.001 |  |  | 0.68 (0.49-0.94) | 0.019 |
|  | Fertility/IVF clinic | 4 | 2,673 | 0.48 (0.07-3.06) | 0.434 |  |  | 1.42 (0.13-15.91) | 0.775 |
|  | Military | 2 | 182,171 | 0.26 (0.03-2.43) | 0.237 |  |  | 0.55 (0.12-2.54) | 0.439 |
|  | Refugee camp | 3 | 638 | 6.86 (0.81-58.10) | 0.077 |  |  | 0.75 (0.10-5.68) | 0.780 |
|  | VCT | 1 | 19,875 | 3.46 (0.15-80.44) | 0.44 |  |  | 5.02 (0.69-36.36) | 0.110 |
|  | Mixed | 4 | 2,335 | 16.49 (3.39-80.22) | <0.001 |  |  | 1.31 (0.46-3.72) | 0.608 |
|  | Unspecified | 44 | 1,552,750 | 1.09 (0.65-1.83) | 0.744 | <0.001 | 10.05 | 1.31 (0.91-1.87) | 0.141 |
| **Study methodology characteristics** | **Sampling methodology** |  |  |  |  |  |  |  |  |
|  | Probability-based | 147 | 355,966 | 1 |  |  |  | 1 | - |
|  | Nonprobability-based | 850 | 47,965,429 | 0.26 (0.20-0.35) | <0.001 |  |  | 0.66 (0.53-0.81) | <0.001 |
|  | Unspecified | 8 | 24,735 | 0.15 (0.04-0.61) | 0.008 | <0.001 | 7.82 | 1.18 (0.44-3.14) | 0.748 |
|  | **Sample size** |  |  |  |  |  |  |  |  |
|  | <100 | 90 | 5,083 | 1 |  |  |  | 1 | - |
|  | ≥100 | 915 | 48,341,047 | 0.21 (0.15-0.29) | <0.001 | <0.001 | 8.31 | 0.61 (0.45-0.84) | 0.002 |
| **Temporal variation** | **Year of data collection^●^** | 1005 | 48,346,130 | 0.95 (0.93-0.96) | <0.001 | <0.001 | 3.67 | 0.96 (0.95-0.97) | <0.001 |
|  | **Year of publication** | 1005 | 48,346,130 | 0.93 (0.92-0.94) | <0.001 | <0.001 | 6.77 | - | - |

Abbreviations: Ab, antibody; ANC, antenatal care; ARR, adjusted relative risk; CI, confidence interval; HCV, hepatitis C virus; IVF, in vitro fertilization; RR, relative risk; VCT, voluntary counselling and testing.

*Afghanistan was chosen as a reference country given the availability of studies in the general population, low prevalence, and being the first country alphabetically to qualify accordingly.

^£^Countries include Iraq, Jordan, Lebanon, Palestine, and Syria.

^**^Countries include Bahrain, Kuwait, Oman, Qatar, Saudi Arabia, and United Arab Emirates.

^∞^Countries include Djibouti, Somalia, Sudan, and Yemen.

^¥^Countries include Algeria, Libya, Mauritania, Morocco, and Tunisia.

**^●^**Due to collinearity between year of data collection and year of publication, the multivariable analysis included only one of these variables, year of data collection.

^a^Variables with a p-value ≤ 0.2 were eligible for inclusion in the multivariable analysis.

^b^The adjusted R-squared for the full model was 65.42%.

**Table S3.** Univariable and multivariable meta-regression analyses for HCV Ab prevalence in all populations, but excluding studies with a sample size <100 participants, in the Middle East and North Africa.

|  |  | **Outcome measures** | **Sample size** | **Univariable analysis** | | | | **Multivariable analysis^b^** | |
| --- | --- | --- | --- | --- | --- | --- | --- | --- | --- |
|  |  | **Total N** | **Total n** | ***RR***  **(95% CI)** | **p-value** | **F p-value**^a^ | **Variance explained R^2^ (%)** | ***ARR***  **(95% CI)** | **p-value** |
| **Population characteristics** | **Population type** |  |  |  |  |  |  |  |  |
|  | General population | 1,074 | 48,985,100 | 1 | - |  |  | 1 | - |
|  | Populations at intermediate risk | 280 | 328,589 | 3.24 (2.64-3.99) | <0.001 |  |  | 2.38 (1.97-2.87) | <0.001 |
|  | High-risk clinical populations | 226 | 114,552 | 15.29 (12.33-18.97) | <0.001 |  |  | 18.75 (15.37-22.86) | <0.001 |
|  | Other special clinical populations | 109 | 96,216 | 6.56 (4.85-8.88) | <0.001 |  |  | 4.81 (3.77-6.14) | <0.001 |
|  | Populations with liver-related conditions | 134 | 125,974 | 16.59 (12.65-21.76) | <0.001 |  |  | 8.79 (7.02-11.00) | <0.001 |
|  | PWID | 87 | 44,746 | 30.94 (22.38-42.77) | <0.001 |  |  | 27.07 (20.34-36.01) | <0.001 |
|  | Mixed populations | 18 | 89,239 | 1.64 (0.81-3.32) | 0.166 | <0.001 | 41.97 | 3.43 (2.00-5.88) | <0.001 |
|  | **Country/subregion** |  |  |  |  |  |  |  |  |
|  | Afghanistan^*^ | 77 | 764,408 | 1 | - |  |  | 1 | - |
|  | Egypt | 303 | 1,747,247 | 6.97 (4.48-10.84) | <0.001 |  |  | 7.02 (5.15-9.55) | <0.001 |
|  | Fertile Crescent^£^ | 313 | 3,785,409 | 0.47 (0.30-0.73) | <0.001 |  |  | 0.49 (0.36-0.67) | <0.001 |
|  | Gulf^**^ | 307 | 21,246,534 | 0.89 (0.57-1.38) | 0.596 |  |  | 0.96 (0.70-1.32) | 0.811 |
|  | Horn of Africa^∞^ | 89 | 93,768 | 0.98 (0.56-1.70) | 0.931 |  |  | 1.06 (0.72-1.55) | 0.773 |
|  | Iran | 333 | 16,222,414 | 1.88 (1.21-2.92) | 0.005 |  |  | 0.66 (0.48-0.89) | 0.008 |
|  | Maghreb^¥^ | 168 | 3,614,483 | 1.35 (0.84-2.18) | 0.213 |  |  | 1.02 (0.73-1.41) | 0.915 |
|  | Pakistan | 338 | 2,350,245 | 4.69 (3.03-7.26) | <0.001 | <0.001 | 22.34 | 3.95 (2.90-5.38) | <0.001 |
|  | **Study site** |  |  |  |  |  |  |  |  |
|  | Blood bank | 490 | 42,178,850 | 1 |  |  |  | 1 | - |
|  | ANC clinic | 56 | 68,479 | 3.42 (2.13-5.51) | <0.001 |  |  | 2.11 (1.53-2.90) | <0.001 |
|  | Central laboratory | 11 | 23,975 | 7.12 (2.58-19.59) | <0.001 |  |  | 2.88 (1.47-5.66) | 0.002 |
|  | Clinical setting | 768 | 1,252,874 | 9.79 (8.08-11.87) | <0.001 |  |  | 1.73 (1.45-2.06) | <0.001 |
|  | Community | 337 | 4,026,291 | 3.61 (2.86-4.57) | <0.001 |  |  | 1.69 (1.43-1.99) | <0.001 |
|  | Fertility/IVF clinic | 4 | 2,673 | 0.49 (0.07-3.22) | 0.455 |  |  | 1.14 (0.30-4.28) | 0.848 |
|  | Military | 2 | 182,171 | 0.27 (0.03-2.58) | 0.253 |  |  | 0.39 (0.09-1.68) | 0.204 |
|  | Prison | 63 | 163,994 | 16.79 (10.89-25.89) | <0.001 |  |  | 4.72 (3.39-6.57) | <0.001 |
|  | Refugee camp | 4 | 2,155 | 2.40 (0.37-15.73) | 0.362 |  |  | 2.04 (0.56-7.37) | 0.277 |
|  | Rehab/drop-in-center | 36 | 16,770 | 42.49 (24.37-74.08) | <0.001 |  |  | 4.91 (3.24-7.46) | <0.001 |
|  | VCT | 8 | 21,637 | 34.30 (10.93-107.58) | <0.001 |  |  | 2.90 (1.31-6.39) | 0.008 |
|  | Mixed | 9 | 3,468 | 16.19 (5.44-48.18) | <0.001 |  |  | 2.92 (1.43-5.97) | 0.003 |
|  | Unspecified | 140 | 1,881,171 | 4.85 (3.53-6.68) | <0.001 | <0.001 | 29.01 | 1.58 (1.25-2.00) | <0.001 |
| **Study methodology characteristics** | **Sampling methodology** |  |  |  |  |  |  |  |  |
|  | Probability-based | 206 | 468,004 | 1 |  |  |  | - | - |
|  | Nonprobability-based | 1,693 | 49,308,336 | 0.78 (0.59-1.04) | 0.093 |  |  | - | - |
|  | Unspecified | 29 | 48,168 | 0.98 (0.44-2.15) | 0.954 | 0.214 | 0.08 | - | - |
| **Temporal variation** | **Year of data collection^●^** | 2622 | 49,824,508 | 0.98 (0.97-0.99) | 0.025 | 0.025 | 0.38 | 0.97 (0.96-0.98) | <0.001 |
|  | **Year of publication** | 2622 | 49,824,508 | 0.98 (0.97-1.00) | 0.004 | 0.004 | 0.18 | - | - |

Abbreviations: Ab, antibody; ANC, antenatal care; ARR, adjusted relative risk; CI, confidence interval; HCV, hepatitis C virus; IVF, in vitro fertilization; PWID, people who inject drugs; RR, relative risk; VCT, voluntary counselling and testing.

*Afghanistan was chosen as a reference country given the availability of studies in the general population, low prevalence, and being the first country alphabetically to qualify accordingly.

^£^Countries include Iraq, Jordan, Lebanon, Palestine, and Syria.

^**^Countries include Bahrain, Kuwait, Oman, Qatar, Saudi Arabia, and United Arab Emirates.

^∞^Countries include Djibouti, Somalia, Sudan, and Yemen.

^¥^Countries include Algeria, Libya, Mauritania, Morocco, and Tunisia.

**^●^**Due to collinearity between year of data collection and year of publication, the multivariable analysis included only one of these variables, year of data collection.

^a^Variables with a p-value ≤ 0.2 were eligible for inclusion in the multivariable analysis.

^b^The adjusted R-squared for the full model was 70.42%.

**Table S3.** Preferred Reporting Items for Systematic Reviews and Meta-analyses (PRIMSA) checklist.

| **Section/topic** | **#** | **Checklist item** | **Reported in main text on** |
| --- | --- | --- | --- |
| **TITLE** | | |  |
| Title | 1 | Identify the report as a systematic review, meta-analysis, or both. | p. 1 |
| **ABSTRACT** | | |  |
| Structured summary | 2 | Provide a structured summary including, as applicable: background; objectives; data sources; study eligibility criteria, participants, and interventions; study appraisal and synthesis methods; results; limitations; conclusions and implications of key findings; systematic review registration number. | p. 2 |
| **INTRODUCTION** | | |  |
| Rationale | 3 | Describe the rationale for the review in the context of what is already known. | p. 3 |
| Objectives | 4 | Provide an explicit statement of questions being addressed with reference to participants, interventions, comparisons, outcomes, and study design (PICOS). | p. 3-4 |
| **METHODS** | | |  |
| Protocol and registration | 5 | Indicate if a review protocol exists, if and where it can be accessed (e.g., Web address), and, if available, provide registration information including registration number. | p. 4-5 |
| Eligibility criteria | 6 | Specify study characteristics (e.g., PICOS, length of follow-up) and report characteristics (e.g., years considered, language, publication status) used as criteria for eligibility, giving rationale. | ^1-8^ |
| Information sources | 7 | Describe all information sources (e.g., databases with dates of coverage, contact with study authors to identify additional studies) in the search and date last searched. | p. 4-5 |
| Search | 8 | Present full electronic search strategy for at least one database, including any limits used, such that it could be repeated. | ^1-8^ |
| Study selection | 9 | State the process for selecting studies (i.e., screening, eligibility, included in systematic review, and, if applicable, included in the meta-analysis). | p. 4-5, ^1-8^ |
| Data collection process | 10 | Describe method of data extraction from reports (e.g., piloted forms, independently, in duplicate) and any processes for obtaining and confirming data from investigators. | p. 4-5, ^1-8^ |
| Data items | 11 | List and define all variables for which data were sought (e.g., PICOS, funding sources) and any assumptions and simplifications made. | ^1-8^ |
| Risk of bias in individual studies | 12 | Describe methods used for assessing risk of bias of individual studies (including specification of whether this was done at the study or outcome level), and how this information is to be used in any data synthesis. | ^1-8^ |
| Summary measures | 13 | State the principal summary measures (e.g., risk ratio, difference in means). | p. 4-5 |
| Synthesis of results | 14 | Describe the methods of handling data and combining results of studies, if done, including measures of consistency (e.g., I^2^) for each meta-analysis. | ^1-8^ |
| Risk of bias across studies | 15 | Specify any assessment of risk of bias that may affect the cumulative evidence (e.g., publication bias, selective reporting within studies). | p. 5-6 |
| Additional analyses | 16 | Describe methods of additional analyses (e.g., sensitivity or subgroup analyses, meta-regression), if done, indicating which were pre-specified. | p. 5-6 |
| **RESULTS** | | |  |
| Study selection | 17 | Give numbers of studies screened, assessed for eligibility, and included in the review, with reasons for exclusions at each stage, ideally with a flow diagram. | ^1-8^ |
| Study characteristics | 18 | For each study, present characteristics for which data were extracted (e.g., study size, PICOS, follow-up period) and provide the citations. | ^1-8^ |
| Risk of bias within studies | 19 | Present data on risk of bias of each study and, if available, any outcome level assessment (see item 12). | ^1-8^ |
| Results of individual studies | 20 | For all outcomes considered (benefits or harms), present, for each study: (a) simple summary data for each intervention group (b) effect estimates and confidence intervals, ideally with a forest plot. | ^1-8^ |
| Synthesis of results | 21 | Present results of each meta-analysis done, including confidence intervals and measures of consistency. | ^1-8^ |
| Risk of bias across studies | 22 | Present results of any assessment of risk of bias across studies (see Item 15). | ^1-8^ |
| Additional analysis | 23 | Give results of additional analyses, if done (e.g., sensitivity or subgroup analyses, meta-regression [see Item 16]). | p. 6-9 |
| **DISCUSSION** | | |  |
| Summary of evidence | 24 | Summarize the main findings including the strength of evidence for each main outcome; consider their relevance to key groups (e.g., healthcare providers, users, and policy makers). | p. 9-12 |
| Limitations | 25 | Discuss limitations at study and outcome level (e.g., risk of bias), and at review-level (e.g., incomplete retrieval of identified research, reporting bias). | p. 11-12 |
| Conclusions | 26 | Provide a general interpretation of the results in the context of other evidence, and implications for future research. | p. 12 |
| **FUNDING** | | |  |
| Funding | 27 | Describe sources of funding for the systematic review and other support (e.g., supply of data); role of funders for the systematic review. | p. 13 |

References

1 Mohamoud, Y. A., Mumtaz, G. R., Riome, S., Miller, D. & Abu-Raddad, L. J. The epidemiology of hepatitis C virus in Egypt: a systematic review and data synthesis. *BMC Infectious Diseases* **13**, 288, doi:10.1186/1471-2334-13-288 (2013).

2 Chemaitelly, H., Chaabna, K. & Abu-Raddad, L. J. The Epidemiology of Hepatitis C Virus in the Fertile Crescent: Systematic Review and Meta-Analysis. *PLOS ONE* **10**, e0135281, doi:10.1371/journal.pone.0135281 (2015).

3 Chemaitelly, H., Mahmud, S., Rahmani, A. M. & Abu-Raddad, L. J. The epidemiology of hepatitis C virus in Afghanistan: systematic review and meta-analysis. *International journal of infectious diseases : IJID : official publication of the International Society for Infectious Diseases* **40**, 54-63, doi:10.1016/j.ijid.2015.09.011 (2015).

4 Fadlalla, F. A., Mohamoud, Y. A., Mumtaz, G. R. & Abu-Raddad, L. J. The epidemiology of hepatitis C virus in the maghreb region: systematic review and meta-analyses. *PloS one* **10**, e0121873, doi:10.1371/journal.pone.0121873 (2015).

5 Chaabna, K., Kouyoumjian, S. P. & Abu-Raddad, L. J. Hepatitis C Virus Epidemiology in Djibouti, Somalia, Sudan, and Yemen: Systematic Review and Meta-Analysis. *PloS one* **11**, e0149966, doi:10.1371/journal.pone.0149966 (2016).

6 Mohamoud, Y. A., Riome, S. & Abu-Raddad, L. J. Epidemiology of hepatitis C virus in the Arabian Gulf countries: Systematic review and meta-analysis of prevalence. *International Journal of Infectious Diseases* **46**, 116-125 (2016).

7 Al Kanaani, Z., Mahmud, S., Kouyoumjian, S. P. & Abu-Raddad, L. J. The epidemiology of hepatitis C virus in Pakistan: systematic review and meta-analyses. *Royal Society open science* **5**, 180257, doi:10.1098/rsos.180257 (2018).

8 Mahmud, S., Akbarzadeh, V. & Abu-Raddad, L. J. The epidemiology of hepatitis C virus in Iran: Systematic review and meta-analyses. *Scientific reports* **8**, 150 (2018).
